# Genetic Variants of lncRNA *GAS5* Are Associated with the Clinicopathologic Development of Oral Cancer

**DOI:** 10.3390/jpm11050348

**Published:** 2021-04-26

**Authors:** Ming-Hong Hsieh, Hsueh-Ju Lu, Chiao-Wen Lin, Chia-Yi Lee, Shang-Jung Yang, Pei-Hsuan Wu, Mu-Kuan Chen, Shun-Fa Yang

**Affiliations:** 1 School of Medicine, Chung Shan Medical University, Taichung 402, Taiwan; mhhpsy@hotmail.com (M.-H.H.); hsuehju0311@gmail.com (H.-J.L.); 2 Department of Psychiatry, Chung Shan Medical University Hospital, Taichung 402, Taiwan; 3 Division of Hematology and Oncology, Department of Internal Medicine, Chung Shan Medical University Hospital, Taichung 402, Taiwan; 4 Institute of Oral Sciences, Chung Shan Medical University, Taichung 402, Taiwan; cwlin@csmu.edu.tw; 5 Department of Dentistry, Chung Shan Medical University Hospital, Taichung 402, Taiwan; 6 Department of Ophthalmology, Show Chwan Memorial Hospital, Changhua 500, Taiwan; ao6u.3msn@hotmail.com; 7 Department of Radiology, Shuang-Ho Hospital, Taipei Medical University and School of Medicine, Zhonghe 235, Taiwan; abyss1989@hotmail.com; 8 College of Medicine, Taipei Medical University, Taipei 110, Taiwan; 9 Department of Otolaryngology-Head and Neck Surgery, Tri-Service General Hospital, Taipei 114, Taiwan; paulownia497@gmail.com; 10 Department of Otorhinolaryngology-Head and Neck Surgery, Changhua Christian Hospital, Changhua 500, Taiwan; 11 Institute of Medicine, Chung Shan Medical University, Taichung 402, Taiwan; 12 Department of Medical Research, Chung Shan Medical University Hospital, Taichung 402, Taiwan

**Keywords:** growth arrest-specific 5, single nucleotide polymorphism, oral cancer, cell differentiation, tumor stage

## Abstract

The long noncoding RNA, Growth arrest-specific 5 (GAS5) plays a crucial role in the development of oral cancer. However, potential genetic variants in *GAS5* that affect the susceptibility and progression of oral cancer have rarely been explored. In this study, two loci of *GAS5* single nucleotide polymorphisms (SNPs) (rs145204276 and rs55829688) were genotyped by using the TaqMan allelic discrimination in 1125 oral cancer patients and 1195 non-oral-cancer individuals. After statistical analyses, the distribution of both the *GAS5* SNP rs145204276 and *GAS5* SNP rs55829688 frequencies were similar between the study and control groups. However, the patients with *GAS5* SNP rs145204276 variants (Ins/Del or Del/Del) showed a higher tendency of moderate to poor cell differentiation of oral cancer (OR: 1.454, 95% CI: 1.041–2.031, *p* = 0.028). Moreover, the *GAS5* SNP rs145204276 variants (Ins/Del or Del/Del) in the non-alcohol-drinking population were associated with significantly advanced tumor stage (OR: 1.500, 95% CI: 1.081–2.081, *p* = 0.015) and larger tumor size (OR: 1.494, 95% CI: 1.076–2.074, *p* = 0.016). Furthermore, individuals with the *GAS5* SNP rs145204276 variant were associated with a higher expression of GAS5 in the GTEx database (*p* = 0.002), and the higher GAS5 level was associated with poor cell differentiation, advanced tumor stage and larger tumor size in head and neck squamous cell carcinoma from the TCGA database (all *p* < 0.05). In conclusion, the *GAS5* SNP rs145204276 variant is related to poor-differentiation cell status in oral cancer. Besides, the presence of the *GAS5* SNP rs145204276 variant is associated with a worse tumor stage and tumor size in oral cancer patients without alcohol drinking.

## 1. Introduction

Oral cancer is one of the most prevalent malignancies throughout the world and leads to significant problems for public health [1,2]. In a previous epidemiological survey, the annual new cases of oral cancer were above 400 thousand worldwide, and a higher incidence was found in western countries and a Southern-Eastern Asia population [3]. The known risk factors for oral cancer include betel nut chewing, alcohol consumption and cigarette smoking [3,4,5,6], and the male-to-female ratio of oral cancer development was estimated as 5.62:1 in Taiwan [7]. The current treatment of oral cancer combines surgical excision, radiotherapy and chemotherapy [1], with five-year survival rates of about 40 to 60 percent [8,9,10]. Recently, the role of target therapy targeting cancer cell markers is under investigation [11].

Similar to other types of cancers, the environment as well as genetic factors will influence the clinical presentation and disease severity of oral cancer [12,13,14,15,16]. For instance, the insulin-like growth factor 2 mRNA-binding protein 2, a protein that contributes to insulin resistance and lipid metabolism, was related to less favorable clinical characters for oral cancer [17]. Additionally, the genetic polymorphism of chemokine receptor-2 gene enhances the susceptibility of oral cancer development [18]. In a survey on the gene other than protein, an early study showed that the single nucleotide polymorphism (SNP) of HOX transcript antisense intergenic RNA (HOTAIR) led to large-size tumors and an elevated risk of lymph node metastasis in non–betel quid chewers [19], implying the relationship between oral cancer and RNA polymorphism.

The Growth arrest-specific 5 (GAS5) is a long non-coding RNA that obtained the name due to the overexpression of itself in the growth-arrested cells [20,21]. Due to the ability of GAS5 to induce apoptosis and suppress tumor growth, the relationship between GAS5 and a wide range of malignancies was evaluated [21,22]. The overexpression of GAS5 can inhibit the proliferation of gastric cancer cells, [23] and a higher GAS5 level may cause the retardation of tumor angiogenesis [24]. On the other hand, the genetic polymorphism of *GAS5* can lead to different effects on tumorigenesis; the SNP rs145204276 del/del genotype of *GAS5* was associated with higher susceptibility of glioma [25]. Since the expression of GAS5 and its SNP influences the clinical characters in many neoplasms, it may also affect the clinical course of oral cancer for each gender, but this has rarely been evaluated in detail.

The purpose of the current study is to evaluate whether the distribution of *GAS5* SNP will affect the clinicopathological characters of oral cancer in a male population. In addition, the distribution of *GAS5* SNP between patients with oral cancer and non-oral-cancer individuals was also analyzed.

## 2. Materials and Methods

### 2.1. Subject Selection

A prospective case-control study was conducted in both the Changhua Christian Hospital and Chung Shan Medical University Hospital, Taichung, Taiwan. Subjects who were (1) diagnosed with oral cancers, (2) of male gender and (3) were followed up with for at least six months in the Changhua Christian Hospital or the Chung Shan Medical University Hospital were recruited as the study group. After the selection, a total of 1125 men with oral cancer were included. For comparison, 1195 gender-matched, cancer-free controls were randomly selected from the Taiwan Biobank Project. The medical charts of these patients were examined, and the demographic data including the age, betel quid chewing condition, cigarette smoking status and alcohol drinking status of each subject were obtained. To analyze the genetic polymorphism of *GAS5*, venous blood drawing was performed for all patients in the Changhua Christian Hospital and Chung Shan Medical University Hospital, and the venous blood samples were preserved in ethylenediaminetetraacetic acid-containing tubes. After the reservation of venous blood samples, each sample was immediately centrifuged and stored in the laboratory refrigerator at approximately −80 degrees Celsius for the analyses. Peripheral blood was collected from each participant after informed consent. The research design was approved by the Institutional Review Board of the Chung Shan Medical University Hospital (CSMUH No: CS15125).

### 2.2. Genomic Extraction and Determination of GAS5 SNP via Real-Time PCR

Two SNPs of *GAS5*, meaning the rs145204276 (Ins/Del) and the rs55829688 (T/C), were chosen since their minor allele frequencies were more than 5 percent, and earlier studies indicated their influence in other cancers [26,27]. The genotyping procedure used in the current study was similar as a previous study [28]; the genome was firstly extracted from the leukocytes in the venous blood from each participant via the QIAamp DNA kits (Qiagen, Valencia, Valencia, CA, USA) according to the manufacturer’s instruction for DNA isolation [29,30]. Then, the isolated DNA was stored at –20 degrees Celsius. After that, the genetic polymorphism concerning both the *GAS5* SNPs rs145204276 and the rs55829688 (T/C) were evaluated via the application of the ABI StepOne Real-Time PCR System (Applied Biosystems, Foster City, CA, USA). Then, the results of the *GAS5* polymorphisms were analyzed by SDS version 3.0 software (Applied Biosystems, Foster City, CA, USA).

### 2.3. Bioinformatics Analysis

To illustrate the relationship between rs145204276 SNP and GAS5 expression, the data from the Genotype-Tissue Expression (GTEx) database were applied to reveal the correlations between rs145204276 SNP and GAS5 expression in esophagus mucosa tissues [31]. Moreover, the correlation between GAS5 expression and the clinical presentations of oral cancers (head and neck squamous cell carcinoma) was demonstrated according to the dataset of The Cancer Genome Atlas (TCGA) [32].

### 2.4. Statistical Analysis

SAS version 9.4 (SAS Institute Inc., Cary, NC, USA) was applied for the statistical analyses in the current study. Descriptive analysis by mean, standard deviation (SD) or percentage was used to show the demographic data between the oral cancer patients and non-oral-cancer individuals. Additionally, the tumor stage, including the tumor, node, metastasis (TNM) status and tumor cell differentiation in the study group, was expressed via descriptive analysis. Then, the Mann-Whitney U test was used to compare the differences in demographic data between the study and control groups. After that, logistic regression was applied to calculate the odds ratio (OR) of the *GAS5* SNP distribution between the study and control groups, while multiple logistic regression was used to yield the adjusted odds ratios (AOR) with correlated 95% confidence intervals (CI) for the two *GAS5* SNPs distribution between the study and control groups, after adjusting for betel nut chewing, alcohol use and tobacco consumption. In addition, the influence of the *GAS5* SNPs frequency on the clinicopathological characteristics of oral cancer was evaluated via logistic regression again for the whole study group, and a subgroup analysis of the oral cancer patients without alcohol use history was conducted with the same method. A *p* value that is less than 0.05 was regarded as statistically significance.

## 3. Results

### 3.1. Basic Characters between the Oral Cancer and Non-Oral Cancer Individuals

The mean age was 55.28 ± 10.98 years old in the study group and 53.91 ± 10.01 years old in the control group without a significant difference (*p* = 0.667) (Table 1). On the other hand, the study group showed higher ratios of betel nut chewing (73.9% versus 16.6%), cigarette smoking (84.7% versus 53.1%) and alcohol drinking (46.8% versus 19.8%) compared to the control group with statistical significance (all *p* < 0.001) (Table 1). The details of cancer subtype and clinical characters of oral cancer in the study group are shown in Table 1.

### 3.2. Distribution of GAS5 SNP Frequencies between the Study and Control Groups

Regarding *GAS5* SNP rs145204276, 59.1% and 58.7% patients in the study and control groups, respectively, expressed the rs145204276 Ins/Del or Del/Del genotypes, and the distribution of *GAS5* SNP rs145204276 frequencies was similar between the study and control groups with similar AOR (Table 2). Besides, the *GAS5* SNP rs55829688 TC + CC genotype was found in 51.9% and 50.2% of patients in the study and control groups, respectively. The distribution of *GAS5* SNP rs55829688 frequencies also showed no significant difference between the two groups concerning the AOR (Table 2).

### 3.3. Distribution Frequency of GAS5 SNP rs145204276 and the Clinicopathological Characteristics of Oral Cancers

Concerning the clinicopathological characteristics of oral cancers with different genotypes of *GAS5* SNP rs145204276, the patients with *GAS5* SNP rs145204276 variants (Ins/Del or Del/Del) showed a higher tendency of moderate to poor cell differentiation of oral cancer (OR: 1.454, 95% CI: 1.041–2.031, *p* = 0.028). Nevertheless, the genotypes of *GAS5* SNP rs145204276 did not influence the status of clinical stage, tumor size, lymph node invasion or distal metastasis (all *p* > 0.05) (Table 3). Moreover, the genotypes of *GAS5* SNP rs55829688 did not influence the clinicopathological characteristics of oral cancer (data not shown). In the subgroup analysis that only enrolled the oral cancer patients without alcohol drinking, the *GAS5* SNP rs145204276 variants (Ins/Del or Del/Del) in this population were associated with significantly advanced tumor stage (OR: 1.500, 95% CI: 1.081–2.081, *p* = 0.015) and larger tumor size (OR: 1.494, 95% CI: 1.076–2.074, *p* = 0.016). The other clinicopathological characteristics of oral cancer, including lymph node invasion, distal metastasis and cell differentiation, did not relate to the presence of *GAS5* SNP rs145204276 variants (all *p* > 0.05) (Table 4).

### 3.4. Relationship among GAS5 rs145204276, GAS5 Expression and Clinical Characters of Head and Neck Cancers from Worldwide Database

**Figure 1:**
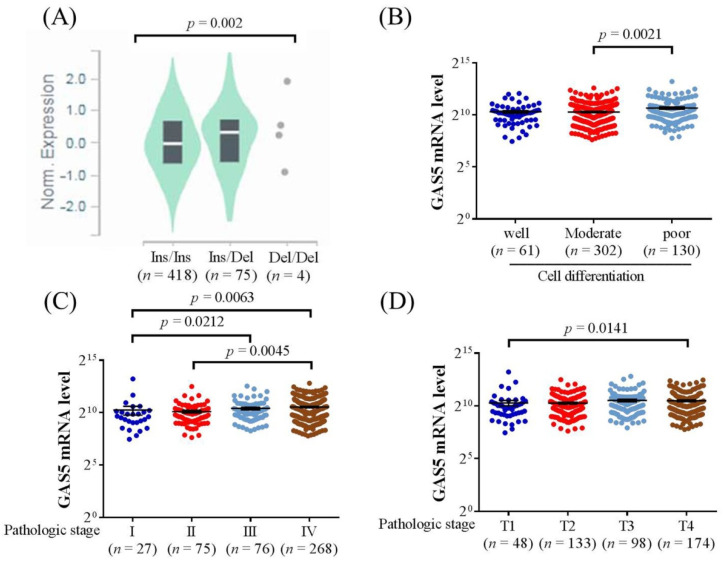
The relationship between growth arrest-specific 5 expression to growth arrest-specific 5 single nucleotide polymorphism rs145204276 and clinical characters of squamous cell carcinoma. (**A**) The relationship between the presence of growth arrest-specific 5 single nucleotide polymorphism rs145204276 (Ins/Ins, *n* = 418; Ins/Del, *n* = 75; Del/Del, *n* = 4) and the growth arrest-specific 5 expression in the esophagus mucosa tissues from Genotype-Tissue Expression database. (**B**) The correlation between the growth arrest-specific 5 mRNA level and the cell differentiation degree of head and neck squamous cell carcinoma from The Cancer Genome Atlas database. (**C**) The correlation between the growth arrest-specific 5 mRNA level and the cancer stage of head and neck squamous cell carcinoma from The Cancer Genome Atlas database. (**D**) The correlation between the growth arrest-specific 5 mRNA level and the tumor size of head and neck squamous cell carcinoma from The Cancer Genome Atlas database.

To strengthen our findings and hypotheses further by incorporating other real-world experiences, the data from the GTEx were obtained and analyzed in the current study. The results showed that individuals with the *GAS5* SNP rs145204276 variant Ins/Del (*n* = 75) or Del/Del (*n* = 4) were associated with a higher expression of GAS5 in the esophagus mucosa tissues with statistical significance (*p* = 0.002) (Figure 1A). However, there was no significant correlation between the *GAS5* SNP rs55829688 and GAS5 expression (data not shown). Furthermore, the data from the TCGA were applied to evaluate the correlation between GAS5 level and head and neck squamous cell carcinoma. The results demonstrated that a higher GAS5 mRNA level was found in the poor-differentiated cancer cells than the moderate-differentiated cancer cells (Figure 1B). Besides, cancer in the stage IV category and T4 stage also showed a higher GAS5 mRNA level compared to the stage I category and T1 stage (*p* = 0.006 and 0.014, respectively) (Figure 1C,D).

## 4. Discussion

In the current study, we found the *GAS5* SNP rs145204276 variants (Ins/Del or Del/Del) are related to a poor-differentiation cell character of oral cancer in a male population. Moreover, the same *GAS5* SNP is correlated with both the advanced tumor stage and larger tumor size of oral cancer in patients without alcohol consumption. The findings of the current study correspond to the results of a real-world database in which the expression of GAS5 is correlated with the *GAS5* SNP rs145204276 variants and severe tumor grading of head and neck squamous cell carcinoma [31].

There are several genetic and environmental confounders that would influence the clinicopathological characteristics of oral cancer [1,3,11,12,33,34,35]. The pro-inflammatory cytokine interleukin 8 and related SNP is associated with the susceptibility of oral squamous cell carcinoma [36]. In addition, the higher Insulin-like growth factor 2 mRNA-binding protein 2 was found in head and neck squamous cell carcinoma and correlated with higher cancer stage and larger tumor size [17], while the presence of *ADAMTS14* SNP rs12774070 was significantly associated with the degree of oral tumor cell differentiation [37]. In addition, gene-environment interactions exist in oral cancer; *CD44* polymorphisms and betel quid chewing or tobacco smoking enhance the susceptibility to oral cancer occurrence [38]. Additionally, for the factor that controls cell growth, the MIR4435-2HG caused the up-regulation of TGF-β1 in oral squamous cell carcinoma [39]. Regarding the aspect of GAS5, it was proven to alter the tumor expression in several types of neoplasms [22,28]. Although the majority of malignancies were suppressed via the higher level of GAS5 [21,23,27], certain cancers benefited from the presence of GAS5 [40]. In previous research, the GAS5 is associated with the progression of glioma [25]. Besides, the *GAS5* SNP rs145204276 variant correlated with a poorer five-year survival rate in uterine cervical cancer [26]. According to the above evidences, the GAS5 could lead to tumor development, and SNP rs145204276 might be related to tumor progression [26,40], and the GAS5 can lead to proliferation and invasion of esophageal cancer, which is anatomically similar to the development site of oral cancer [41]. On the other hand, the presence of GAS5 could suppress tumor invasion and proliferation via the miR-21/PTEN axis in the oral squamous cell carcinoma [42]. Moreover, two *GAS5* SNPs, which included rs2067079 and rs6790, were associated with a higher possibility of toxic reaction after platinum-based concurrent chemoradiotherapy in patients with nasopharyngeal carcinoma [43]. The above evidence suggests a complex role of GAS5 in the clinical course of oral cancer rather than an absolutely positive or negative role, and such an effect may be altered by specified SNPs and environment factors since both of them can influence the clinical presentation of oral cancer [37,38,44]. Consequently, it is possible that the SNP of *GAS5* may be associated with the development of oral cancer in a specific gender, which was partially supported by the findings of the current study.

The expression of the *GAS5* SNP rs145204276 variant (Ins/Del or Del/Del) is correlated with the poor-differentiation cell type of oral cancer for males in the current study. To our knowledge, this is a preliminary finding that was seldom evaluated elsewhere. A previous study that surveyed a similar ethnicity showed a lower level of GAS5 in the oral squamous cell carcinoma [45]. However, the age and the gender distribution in that study were unknown, so gender may have an interaction effect on the risk factor of cancer [46]. The *GAS5* SNP rs145204276 Del variant increases the expression of GAS5 mRNA, which proven in the previous research [47], and the higher concentration of GAS5 may have a prominent effect on the development of oral cancer. Given the fact that the GAS5 has a different effect, either positive or negative, on the tumor progression, invasion and response to treatment in various types of cancers [23,25,40,48], our findings suggest that the presence of the *GAS5* SNP rs145204276 variant is a positive factor for severe oral cancer in at least a male population, in contrast to the protective effect for oral cancer in a previous study [42]. The previous study only surveyed the protective effect and related pathway of the general GAS5 phenotype against oral squamous cell carcinoma [42], while certain SNPs may change the effect of GAS5 on oral cancer. Still, the exact mechanism of this phenomenon needs further investigation.

In the subgroup analysis of the oral cancer population without alcohol drinking, the presence of the *GAS5* SNP rs145204276 variant is correlated with the advanced cancer stage and larger tumor size. This is in contrast to the results in the whole oral cancer population in which the above two characters did not show significant differences. The possible explanation is that the alcohol-drinking patients have some genetic variations that influence the susceptibility of tumor progression or GAS5 expression. In a previous study, the adulthood patients that did consume alcohol showed a significant correlation with the polymorphisms in 5HTT, DAT1, DRD4, DRD2 and MAOA [49]. Besides, the deficiency of relaxin-3 was found to increase the alcohol consumption in mice significantly [50]. However, these genes were not tested in the current study; thus, the possible interaction between them and *GAS5* SNP needs further research to confirm. On the other hand, the severity of lymph node invasion and distal metastasis did not alter with different *GAS5* SNP rs145204276 in both the whole group and subgroup analysis, which may indicate the universally minimal effects of *GAS5* SNP in the two clinical characters for oral cancer.

Regarding the real-world cancer database, both the GTEx and TCGA found that a higher expression of GAS5 is associated with the alteration of head and neck tissues [31]. These findings correspond to the results in the current study that the expression of *GAS5* SNP may lead to worse clinicopathological characteristics of oral cancer and the adjunct tissue. The *GAS5* SNP rs145204276 is related to the higher level of GAS5 expression in the GTEx database, which was also proven in previous study [47]. Moreover, the advanced tumor stage, larger tumor size and poor-differentiation cell status were observed in head and neck squamous cell carcinoma according to the database of TCGA, and the current study also found such a relationship between the *GAS5* SNP rs145204276 and the above clinicopathological characteristics. The similar outcomes in our study and the database of TCGA should further strengthen the relationship between *GAS5* SNP rs145204276 and the three clinicopathological characteristics of oral cancer, which may be because of a carcinogenic effect of *GAS5* SNP rs145204276 [51]. Concerning ethnicity, the population of TCGA comes from all over the world while the study population of the current study is mostly Han Taiwanese. Additionally, the TCGA enrolled both genders while the current study only considered the male population. Such differences may lead to inconsistencies in the significant influence of *GAS5* SNP rs145204276 on oral cancers between the current study and the results of TCGA.

Regarding the differences between the study group and the control group in the current study, no difference of *GAS5* SNP distribution frequency was found between the two groups for either *GAS5* SNP rs145204276 or *GAS5* SNP rs55829688. This phenomenon may imply that the *GAS5* polymorphism is not a risk factor for oral cancer development in a normal population, but rather a predisposing factor for tumor progression in those with pre-existing oral cancer. Interestingly, the numbers of *GAS5* SNP variant types, whether in rs145204276 or rs55829688, were more than the *GAS5* SNP wild type in both the study and control groups, which indicates that the etiology needs further study. For the demographic characteristics between the two groups, age was similar between the study and control groups since we chose the patients with similar age as the control group. The ratios of other demographic data, including betel nut chewing, cigarette consumption and alcohol drinking, were all higher in the study group compared to the control group. The reason may be that all three demographic data are prominent risk factors for oral cancer [7].

There are still some limitations in the current study. Firstly, the current study is not a double-blind, randomized control trial; thus, some bias may exist. Second, we did not follow up with the patients with an adequate period to observe the clinical course of patients with different *GAS5* polymorphism. In addition, certain diseases such as periodontal disease and chronic Porphyromonas gingivalis are risk factors for the progression of oral cancer [52,53], but we did not enroll them in the multivariable analysis. Finally, oral cancer has several subtypes such as squamous cell carcinomas and mucoepidermoid carcinoma [54,55], but the current study failed to analyze them separately.

## 5. Conclusions

In conclusion, the presence of the *GAS5* SNP rs145204276 variant is correlated with a poor-differentiation cell status in oral cancer in males. Furthermore, a worse tumor stage and tumor size was found in the non-alcohol-drinking group with oral cancer and the *GAS5* SNP rs145204276 variant. The above findings might be applied as an evaluation tool for the disease progression of oral cancer in clinical practice for male populations. Further randomized-controlled trials to evaluate whether the *GAS5* SNP rs145204276 variant will affect the treatment outcome of oral cancer are mandatory.

## Figures and Tables

**Table 1:** The distributions of demographical characteristics in 1195 controls and 1125 male patients with oral cancer.

Variables	Controls (*n* = 1195)	Patients (*n*= 1125)	*p* Value
Age (yrs)	53.91 ± 10.01	55.28 ± 10.98	
<55	564 (47.2%)	541 (48.1%)	*p* = 0.667
≥55	631 (52.8%)	584 (51.9%)	
Betel nut chewing			
No	997 (83.4%)	294 (26.1%)	
Yes	198 (16.6%)	831 (73.9%)	*p* < 0.001 *
Cigarette smoking			
No	560 (46.9%)	172 (15.3%)	
Yes	635 (53.1%)	953 (84.7%)	*p* < 0.001 *
Alcohol drinking			
No	958 (80.2%)	598 (53.2%)	
Yes	237 (19.8%)	527 (46.8%)	*p* < 0.001 *
Cancer location			
Buccal mucosa		398 (35.4%)	
Tongue		356 (31.6%)	
Gingiva		111 (9.9%)	
Lip		57 (5.1%)	
Others		203 (18.0%)	
Stage			
I + II		529 (47.0%)	
III + IV		596 (53.0%)	
Tumor T status			
T1 + T2		582 (51.7%)	
T3 + T4		543 (48.3%)	
Lymph node status			
N0		745 (66.2%)	
N1 + N2 + N3		380 (33.8%)	
Metastasis			
M0		1114 (99.0%)	
M1		11 (1.0%)	
Cell differentiation			
Well differentiated		162 (14.4%)	
Moderately or poorly differentiated		963 (85.6%)	

N: number. * denotes statistically significant between the study and control groups.

**Table 2:** Odds ratio (OR) and 95% confidence interval (CI) of oral cancer associated with *GAS5* genotypic frequencies.

Variables	Controls (*n* = 1195) N (%)	Patients (*n* = 1125) N (%)	OR (95% CI)	AOR (95% CI)
**rs145204276**				
Ins/Ins	493 (41.3%)	460 (40.9%)	1.000 (reference)	1.000 (reference)
Ins/Del	543 (45.4%)	526 (46.8%)	1.038 (0.872–1.236)	1.073 (0.866–1.331)
Del/Del	159 (13.3%)	139 (12.3%)	0.937 (0.722–1.216)	0.986 (0.716–1.358)
Ins/Del + Del/Del	702 (58.7%)	665 (59.1%)	1.015 (0.860–1.198)	1.054 (0.859–1.292)
**rs55829688**				
TT	595 (49.8%)	541 (48.1%)	1.000 (reference)	1.000 (reference)
TC	473 (39.6%)	470 (41.8%)	1.093 (0.919–1.299)	1.077 (0.870–1.332)
CC	127 (10.6%)	114 (10.1%)	0.987 (0.747–1.304)	1.022 (0.726–1.440)
TC + CC	600 (50.2%)	584 (51.9%)	1.070 (0.910–1.260)	1.065 (0.872–1.302)

N: number. OR: odds ratio. AOR: adjusted odds ratio.

**Table 3:** Clinical statuses and *GAS5* rs145204276 genotype frequencies in oral cancer.

Variables	*GAS5* (rs145204276)			
	Ins/Ins (%) (*n* = 460)	Ins/Del + Del/Del (%) (*n* = 665)	OR (95% CI)	*p* Value
**Clinical Stage**				
Stage I/II	231 (50.2%)	298 (44.8%)	1.00	*p* = 0.074
Stage III/IV	229 (49.8%)	367 (55.2%)	1.242 (0.979–1.577)	
**Tumor size**				
≤T2	245 (53.3%)	337 (50.7%)	1.00	*p* = 0.394
>T2	215 (46.7%)	328 (49.3%)	1.109 (0.874–1.407)	
**Lymph node metastasis**				
No	310 (67.4%)	435 (65.4%)	1.00	*p* = 0.490
Yes	150 (32.6%)	230 (34.6%)	1.093 (0.849–1.406)	
**Distant metastasis**				
No	453 (98.5%)	661 (99.4%)	1.00	*p* = 0.123
Yes	7 (1.5%)	4 (0.6%)	0.392 (0.114–1.346)	
**Cell differentiation**				
well	79 (17.2%)	83 (12.5%)	1.00	*p* = 0.028 *
Moderate/poor	381 (82.8%)	582 (87.5%)	1.454 (1.041–2.031)	

* denotes significant difference of clinicopathological characteristics between the different GAS5 rs145204276 genotypes.

**Table 4:** Clinical statuses and *GAS5* rs145204276 genotype frequencies in oral cancer among 598 non-drinkers.

Variables	*GAS5* (Rs145204276)			
	Ins/Ins (%) (*n* = 245)	Ins/Del + Del/Del (%) (*n* = 353)	OR (95% CI)	*p* Value
**Clinical Stage**				
Stage I/II	133 (54.3%)	156 (44.2%)	1.00	*p* = 0.015 *
Stage III/IV	112 (45.7%)	197 (55.8%)	1.500 (1.081–2.081)	
**Tumor size**				
≤T2	139 (56.7%)	165 (46.7%)	1.00	*p* = 0.016 *
>T2	106 (43.3%)	188 (53.3%)	1.494 (1.076–2.074)	
**Lymph node metastasis**				
No	170 (69.4%)	241 (68.3%)	1.00	*p* = 0.772
Yes	75 (30.6%)	112 (31.7%)	1.053 (0.741–1.498)	
**Distant metastasis**				
No	243 (99.2%)	352 (99.7%)	1.00	*p* = 0.364
Yes	2 (0.8%)	1 (0.3%)	0.345 (0.031–3.828)	
**Cell differentiation**				
well	42 (17.1%)	45 (12.7%)	1.00	*p* = 0.134
Moderate/poor	203 (82.9%)	308 (87.3%)	1.416 (0.897–2.235)	

* denotes significant difference of clinicopathological characteristics between the different GAS5 rs145204276 genotypes.

## Data Availability

The datasets generated for this study are available on request to the corresponding authors or in the TCGA databases.
